# Crystal structure and Hirshfeld surface analysis of (*Z*)-*N*-{chloro­[(4-ferrocenylphen­yl)imino]­meth­yl}-4-ferrocenylaniline *N*,*N*-di­methyl­formamide monosolvate

**DOI:** 10.1107/S2056989024001002

**Published:** 2024-02-02

**Authors:** Riham Sghyar, Abdeslem Bentama, Amal Haoudi, Ahmed Mazzah, Joel T. Mague, Tuncer Hökelek, El Mestafa EL Hadrami, Nada Kheira Sebbar

**Affiliations:** a Laboratory of Applied Organic Chemistry, Sidi Mohamed Ben Abdellah University, Faculty of Science And Technology, Road Immouzer, BP 2202 Fez, Morocco; b Science and Technology of Lille USR 3290, Villeneuve d’ascq cedex, France; c Department of Chemistry, Tulane University, New Orleans, LA 70118, USA; d Department of Physics, Hacettepe University, 06800 Beytepe, Ankara, Türkiye; e Laboratory of Organic and Physical Chemistry, Applied Bioorganic Chemistry Team, Faculty of Sciences, Ibnou Zohr University, Agadir, Morocco; f Laboratory of Plant Chemistry, Organic and Bioorganic Synthesis, Faculty of Sciences, Mohammed V University in Rabat, 4 Avenue Ibn Battouta BP 1014 RP, Rabat, Morocco; University of Hyogo, Japan

**Keywords:** crystal structure, ferrocene, carbamidic chloride, hydrogen bond, C—H⋯π(ring) inter­actions

## Abstract

The title mol­ecule, [Fe_2_(C_5_H_5_)_2_(C_23_H_17_ClN_2_)]·C_3_H_7_NO, is twisted end to end and the central N/C/N unit is disordered. In the crystal, several C—H⋯π(ring) inter­actions lead to the formation of layers, which are connected by further C—H⋯π(ring) inter­actions. A Hirshfeld surface analysis of the crystal structure indicates that the most important contributions for the crystal packing are from H⋯H (60.2%) and H⋯C/C⋯H (27.0%) inter­actions. Hydrogen bonding, C—H⋯π(ring) inter­actions and van der Waals inter­actions dominate the crystal packing.

## Abstract 2

The mol­ecule of the title compound is twisted end to end so that the ferrocenyl groups are nearly perpendicular to one another. The central N/C/N unit is disordered. In the crystal, several C—H⋯π(ring) inter­actions lead to the formation of layers parallel to (010), which are connected by further C—H⋯π(ring) inter­actions.

## 1. Chemical context

Organometallic compounds have been studied for almost 250 years and have proved to be bioactive mol­ecules with a wide range of applications (Krause *et al.*, 2012 ▸; Parveen *et al.*, 2019 ▸; Li *et al.*, 2008 ▸). They are characterized by their metal–carbon covalent bonds as well as their kinetic stability, non-chargeability, lipophilicity, and low metal oxidation states (Herrmann, 1988 ▸; Alama *et al.*, 2009 ▸). A number of organometallic compounds are useful starting reagents for organic and organometallic synthesis. Metallocenes are an important and well-known class of organometallic compounds that offer new possibilities in the design of catalytic, biosensing, and medicinal compounds (Gasser *et al.*, 2011 ▸; Gasser & Metzler-Nolte, 2012 ▸; Ong & Gasser, 2020 ▸). Their chemical richness is caused by the variation in electron density in the valence shell. Ferrocene, one of the most prominent metallocene derivatives, is a fascinating target in a variety of fields, including electrochemistry, biochemistry, and drug design (Togni, 1996 ▸; Tsukazaki *et al.*, 1996 ▸; Nishibayashi *et al.*, 1996 ▸) and mediators of protein redox reactions (Dai *et al.*, 2007 ▸). Due to the chemical richness of the iron(II) center, its stability in aqueous and aerobic environments and its aromatic properties, ferrocene has attracted considerable inter­est (Ibrahim, 2001 ▸). In addition to possessing a wide range of derivatives, these compounds are easily oxidized. Ferrocene derivatives have been reported to have anti­tumor, anti­malarial, anti­convulsant, anti­oxidant, anti­microbial and DNA-cleaving activities among their biological activities, and have attracted particular attention as anti­tumor and anti­malarial agents including the drugs tamoxifen, ferroquine and ferrocifen (Top *et al.*, 2003 ▸). These drugs are excellent preventive agents against cancer and malaria, and their biological uses have been the subject of much research. The derivatization of ferrocene has been extensively studied (Rehmani *et al.*, 2010 ▸). Amines, carbonyls and carb­oxy­lic acid functionalities can be introduced to derivatize ferrocene (Langeroodi, 2010 ▸). Ferrocenyl aniline can be synthesized by reducing nitro­phenyl ferrocene. There is an inter­mediary in the synthesis of ferrocene-containing liquid crystals, ferrocene-containing Schiff bases. In our research on the development of new substituted ferrocenyl derivatives, we synthesized *N*,*N*-bis­(4-ferrocenylphen­yl)carbamimidic dichloride by reacting 4-ferrocenyl aniline with (4-ferrocenylphen­yl)carbonimidic dichloride with potassium carbonate as a base and tetra­butyl­ammonium bromide as a catalyst. In this paper, we present the synthesis and detailed examination of the mol­ecular and crystal structures of the title compound, including by Hirshfeld surface analysis. 
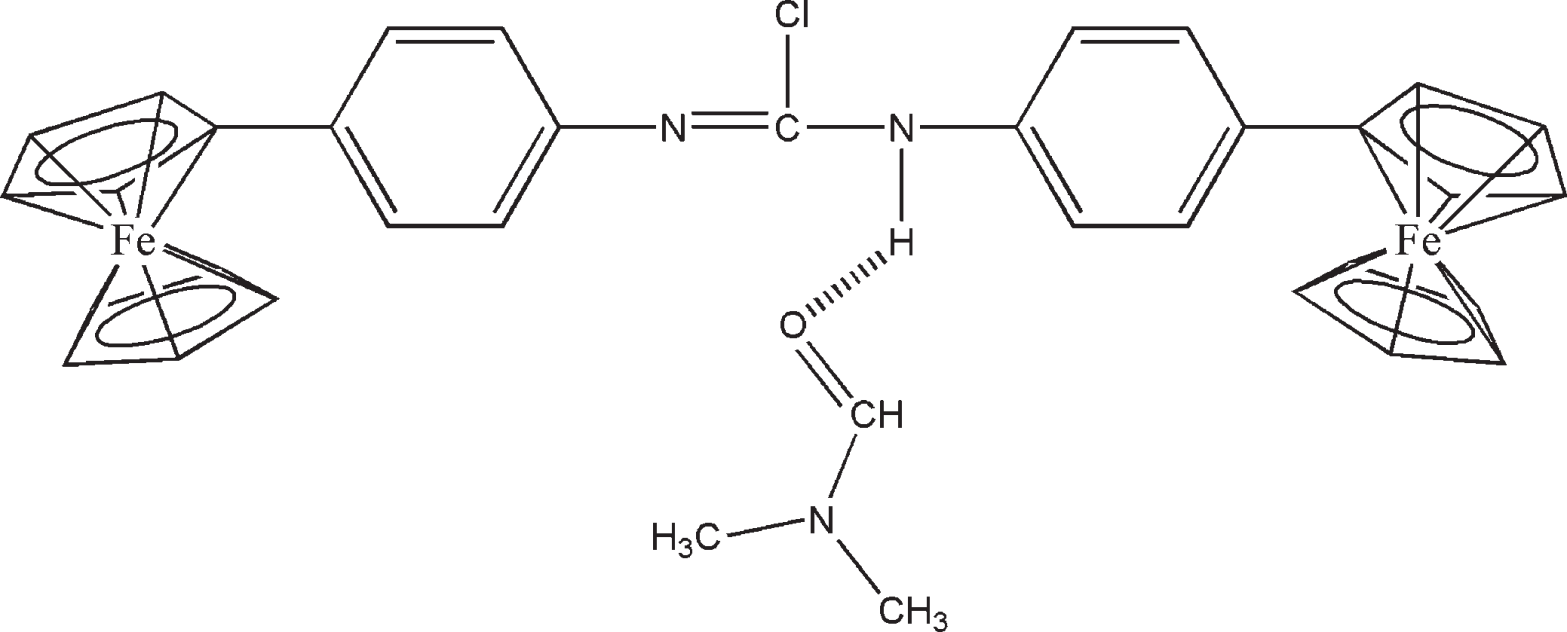


## 2. Structural commentary

**Figure 1:**
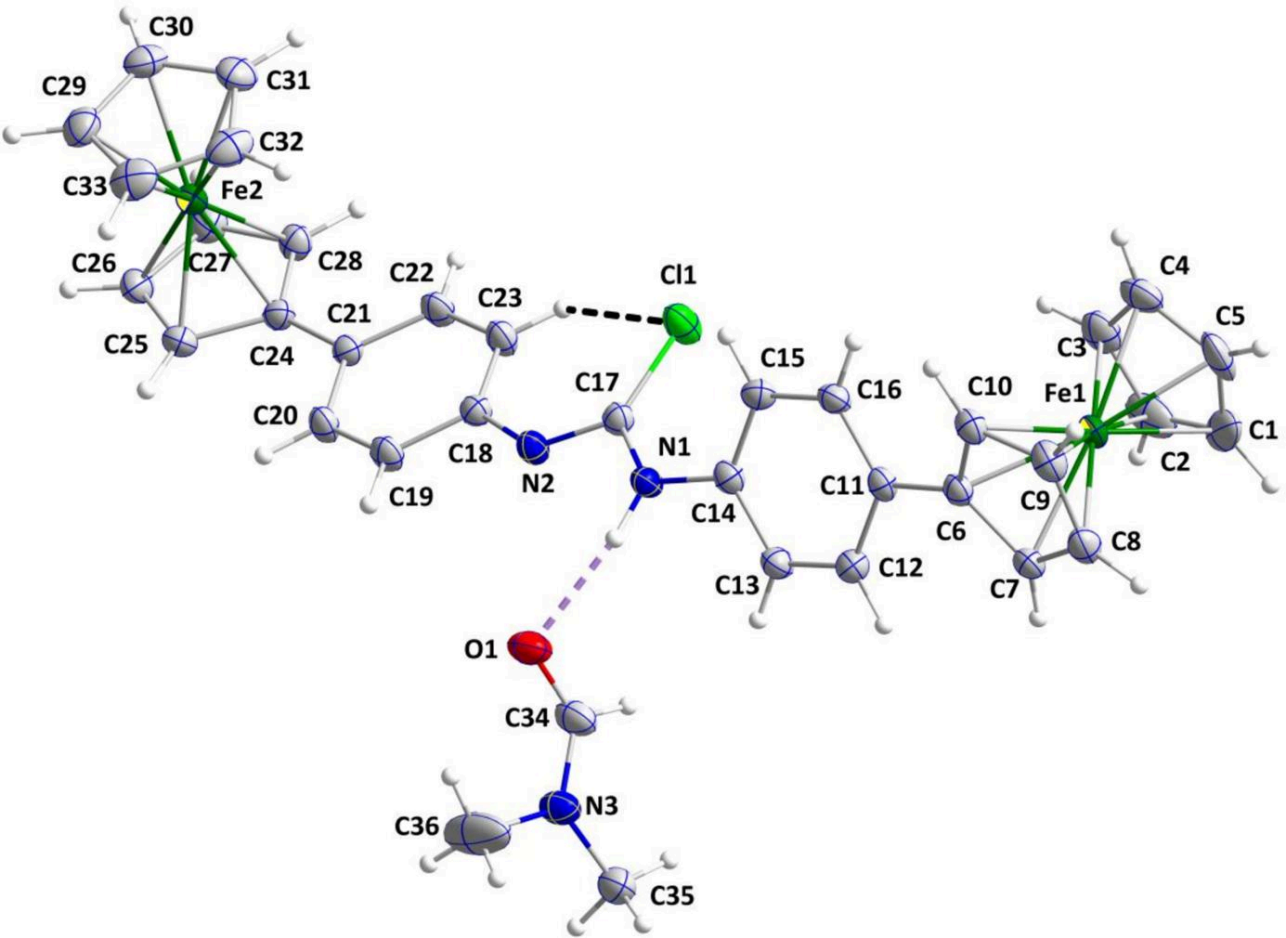
The title mol­ecule with the labeling scheme and 50% probability ellipsoids. Only one component of the disordered N—H group is shown. The C—H⋯Cl and N—H⋯O hydrogen bonds are depicted, respectively, by black and violet dashed lines.

In the crystal, the mol­ecule is disordered in essentially equal amounts such that a hydrogen atom appears on both N1 and N2 and the C17—N1 and C17—N2 distances appear equivalent at 1.365 (3) and 1.366 (3) Å, respectively. The ferrocenyl groups are nearly perpendicular to one another as indicated by the dihedral angle of 82.05 (9)° between the C1–C5 and C29–C33 cyclo­penta­dienyl rings. The cyclo­penta­dienyl rings attached to Fe1 are parallel within experimental error [dihedral angle = 0.14 (18)°] while those attached to Fe2 are not [dihedral angle = 2.03 (19)°]. The mol­ecule is twisted along its length (Fig. 1 ▸), as indicated by the dihedral angles listed in Table 1 ▸. The smaller values for the last two entries in the table are due, in part, to the intra­molecular C23—H23⋯Cl1 hydrogen bond (Table 2 ▸). With the exception of the two C—N distances affected by the disorder, all bond distances and inter­bond angles appear as expected for the given formulation.

## 3. Supra­molecular features

**Figure 2:**
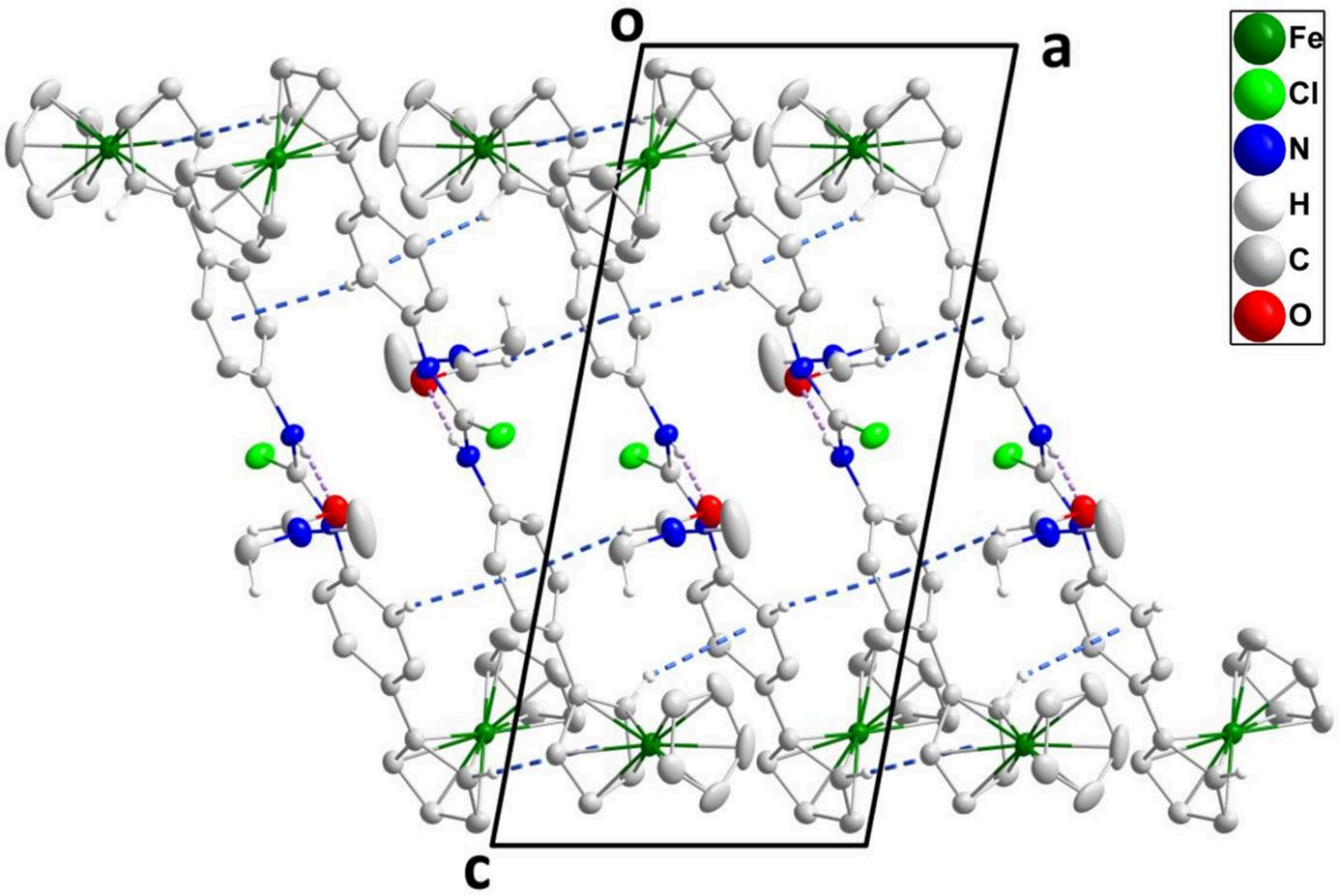
Packing viewed along the *b*-axis direction with N—H⋯O hydrogen bonds and C—H⋯π(ring) inter­actions depicted, respectively, by violet and blue dashed lines.

In the crystal, the DMF solvent mol­ecule is bound to the main mol­ecule by an N1—H1⋯O1 hydrogen bond and these units are formed into corrugated layers parallel to (010) by C7—H7⋯*Cg*6, C19—H19⋯*Cg*5, C25—H25⋯*Cg*2 and C34—H34⋯*Cg*5 inter­actions, while the layers are connected by C35—H35*C*⋯*Cg*4 inter­actions (Table 2 ▸ and Fig. 2 ▸) where *Cg*2, *Cg*4, *Cg*5 and *Cg*6 are the centroids of the C6–C10, C29–C23 C11–C16 and C18–C23 rings, respectively.

## 4. Hirshfeld surface analysis

**Figure 3:**
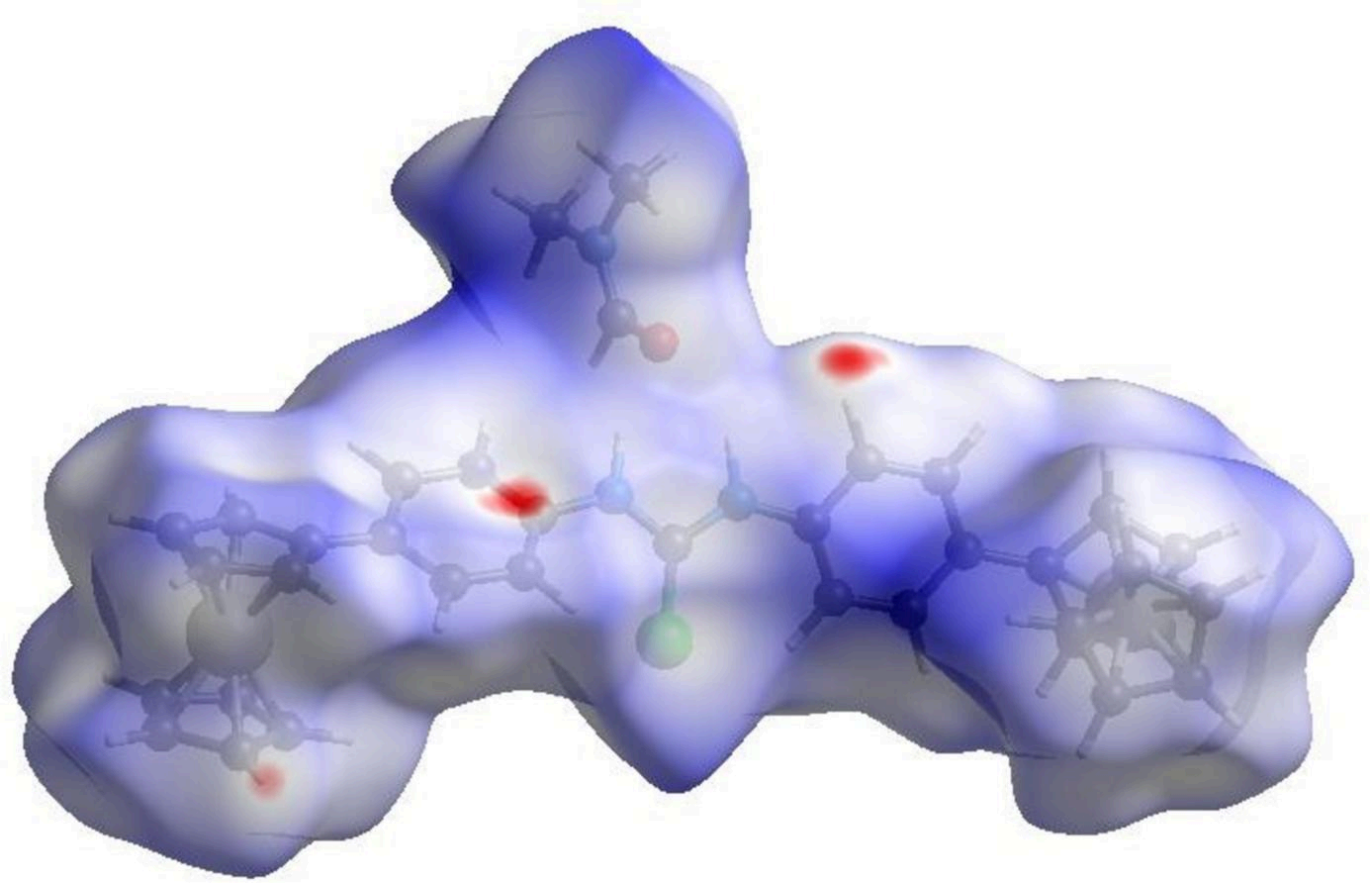
View of the three-dimensional Hirshfeld surface of the title compound plotted over *d*_norm_.

**Figure 4:**
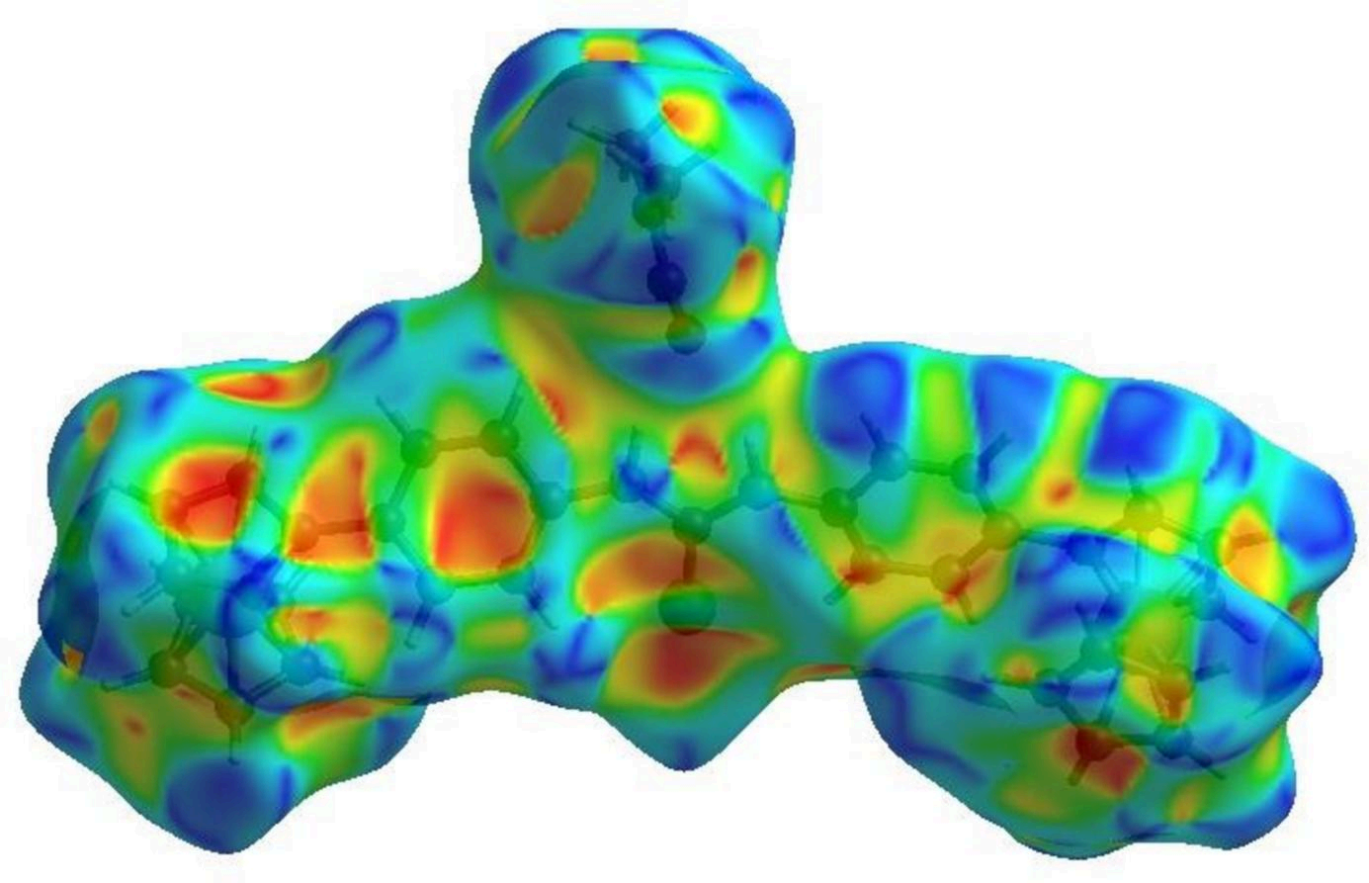
Hirshfeld surface of the title compound plotted over shape-index.

In order to visualize the inter­molecular inter­actions in the crystal of the title compound, a Hirshfeld surface (HS) analysis (Hirshfeld, 1977 ▸; Spackman & Jayatilaka, 2009 ▸) was carried out using *Crystal Explorer 17.5* (Turner *et al.*, 2017 ▸). In the HS plotted over *d*_norm_ (Fig. 3 ▸), the white surface indicates contacts with distances equal to the sum of van der Waals radii, and the red and blue colors indicate distances shorter (in close contact) or longer (distinct contact) than the sum of van der Waals radii, respectively (Venkatesan *et al.*, 2016 ▸). The bright-red spots indicate the respective donors (C14) and/or acceptors (H5 and H19). The shape-index of the HS is a tool to visualize the π–π stacking by the presence of adjacent red and blue triangles; if there are no adjacent red and/or blue triangles, then there are no π–π inter­actions. Fig. 4 ▸ clearly suggests that there are no π–π inter­actions present.

**Figure 5:**
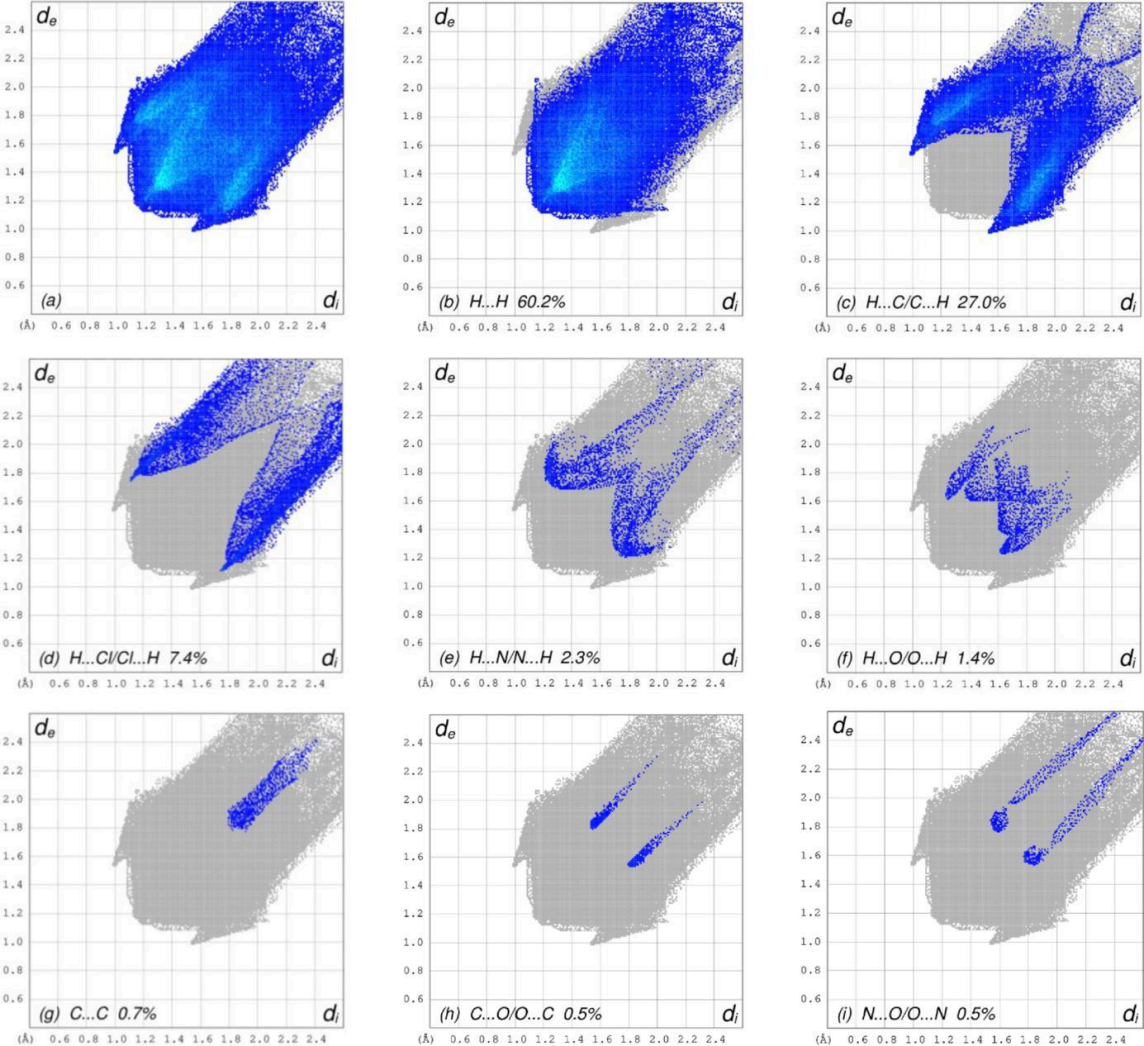
The full two-dimensional fingerprint plots for the title compound, showing (*a*) all inter­actions, and delineated into (*b*) H⋯H, (*c*) H⋯C/C⋯H, (*d*) H⋯Cl/Cl⋯H, (*e*) H⋯N/N⋯H, (*f*) H⋯O/O⋯H, (*g*) C⋯C, (*h*) C⋯O/O⋯C and (*i*) N⋯O/O⋯N inter­actions. The *d*_i_ and *d*_e_ values are the closest inter­nal and external distances (in Å) from given points on the Hirshfeld surface.

**Figure 6:**
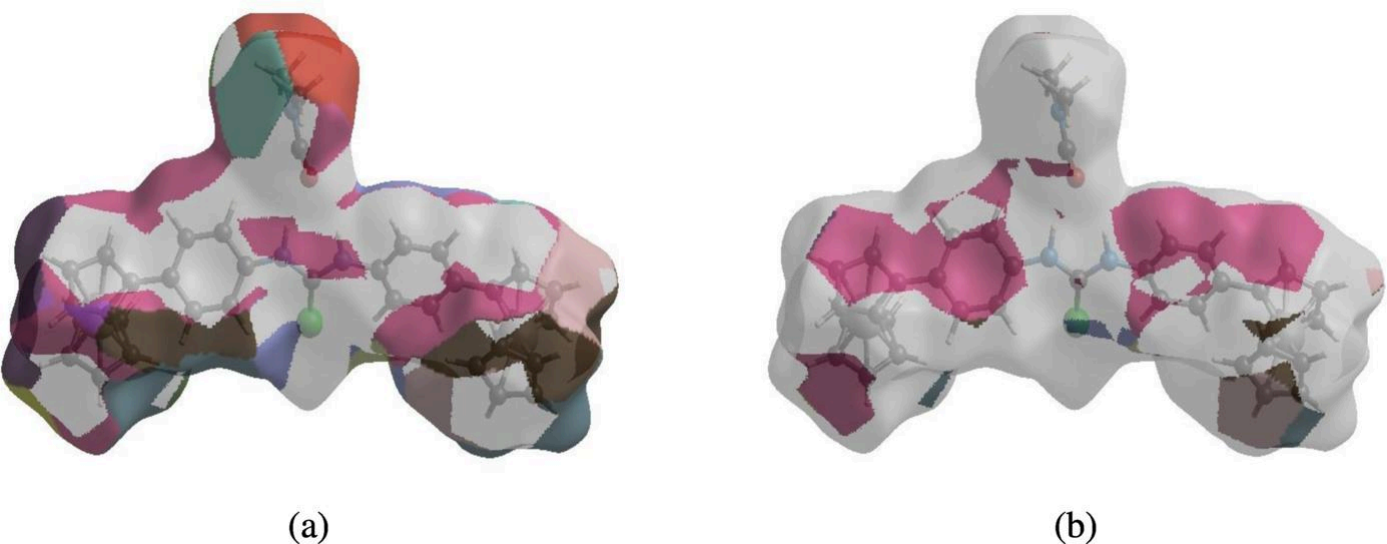
The Hirshfeld surface representations as fragment patches plotted onto the surface for (*a*) H⋯H and (*b*) H⋯C/C⋯H inter­actions.

The overall two-dimensional fingerprint plot is shown in Fig. 5 ▸ *a*, and those delineated into H⋯H, H⋯C/C⋯H, H⋯Cl/Cl⋯H, H⋯N/ N⋯H, H⋯O/O⋯H, C⋯C, C⋯O/O⋯C and N⋯O/O⋯N (McKinnon *et al.*, 2007 ▸) are illustrated in Fig. 5 ▸ *b*–*i*, respectively, together with their relative contributions to the Hirshfeld surface. The most abundant inter­action is H⋯H, contributing 60.2% to the overall crystal packing, which is reflected in Fig. 5 ▸ *b* as the widely scattered points of high density due to the large hydrogen content of the mol­ecule with the tip at *d*_e_ = *d*_i_ = 1.16 Å. As a result of the presence of C—H⋯π inter­actions, the H⋯C/C⋯H contacts contribute 27.0% to the overall crystal packing and are shown in Fig. 5 ▸ *c* with the tips at *d*_e_ + *d*_i_ = 2.51 Å. The pair of characteristic wings in the fingerprint plot delineated into H⋯Cl/Cl⋯H contacts (Fig. 5 ▸ *d*) with the tips at *d*_e_ + *d*_i_ = 2.86 Å contribute 7.4% to the HS. The pair of wings in the fingerprint plot delineated into H⋯N/N⋯H contacts (Fig. 5 ▸ *e*) with a 2.3% contribution to the HS is seen with the tips at *d*_e_ + *d*_i_ = 2.98 Å while the H⋯O/O⋯H (Fig. 5 ▸ *f*) contacts with a 1.4% contribution to the HS are viewed as pairs of wings with the tips at *d*_e_ + *d*_i_ = 2.86 Å and *d*_e_ + *d*_i_ = 3.00Å for the long and short ones, respectively. Finally, the C⋯C (Fig. 5 ▸ *g*), C⋯O/O⋯C (Fig. 5 ▸ *h*) and N⋯O/O⋯N (Fig. 5 ▸ *i*) contacts with 0.7%, 0.5% and 0.5% contributions, respectively, to the HS have very low distributions of points. The Hirshfeld surface representations as fragment patches plotted onto the surface are shown for the H⋯H and H⋯C/C⋯H inter­actions in Fig. 6 ▸ *a*–*b*, respectively. The Hirshfeld surface analysis confirms the importance of H-atom contacts in establishing the packing. The large number of H⋯H and H⋯C/C⋯H inter­actions suggest that van der Waals inter­actions and hydrogen bonding play the major roles in the crystal packing (Hathwar *et al.*, 2015 ▸).

## 5. Database survey

A survey of the Cambridge Structural Database (CSD version, updated to November 2023; Groom *et al.*, 2016 ▸) with the search fragment I (*R* = *R*′ = nothing) yielded five hits, all of which contain only one ferrocenyl group and the first four have a *trans* disposition of *R* and *R*′. These structures include ones with *R* = 2-ClC_6_H_4_NH, *R*′ = PhC(=O) (DEZHUN; Gul *et al.*, 2013*a* ▸); *R* = 3-NO_2_-4-ClC_6_H_3_NH; *R*′ = 3-ClC_6_H_4_C(=O) (JARZUB; Ozdemir, 2021 ▸); R = 3,4-Cl_2_C_6_H_3_NH, *R*′ = 3-ClC_6_H_4_C(=O) (NIKQOP; Gul *et al.*, 2013*b* ▸); *R* = 3-CF_3_C_6_H_4_NH, *R*′ = PhC(=O) (QAGTEA; Gul *et al.*, 2014 ▸) and *R* = *p*-tolNH, *R*′ = PhC(=O) (QAHWAZ; Gul *et al.*, 2014 ▸). 
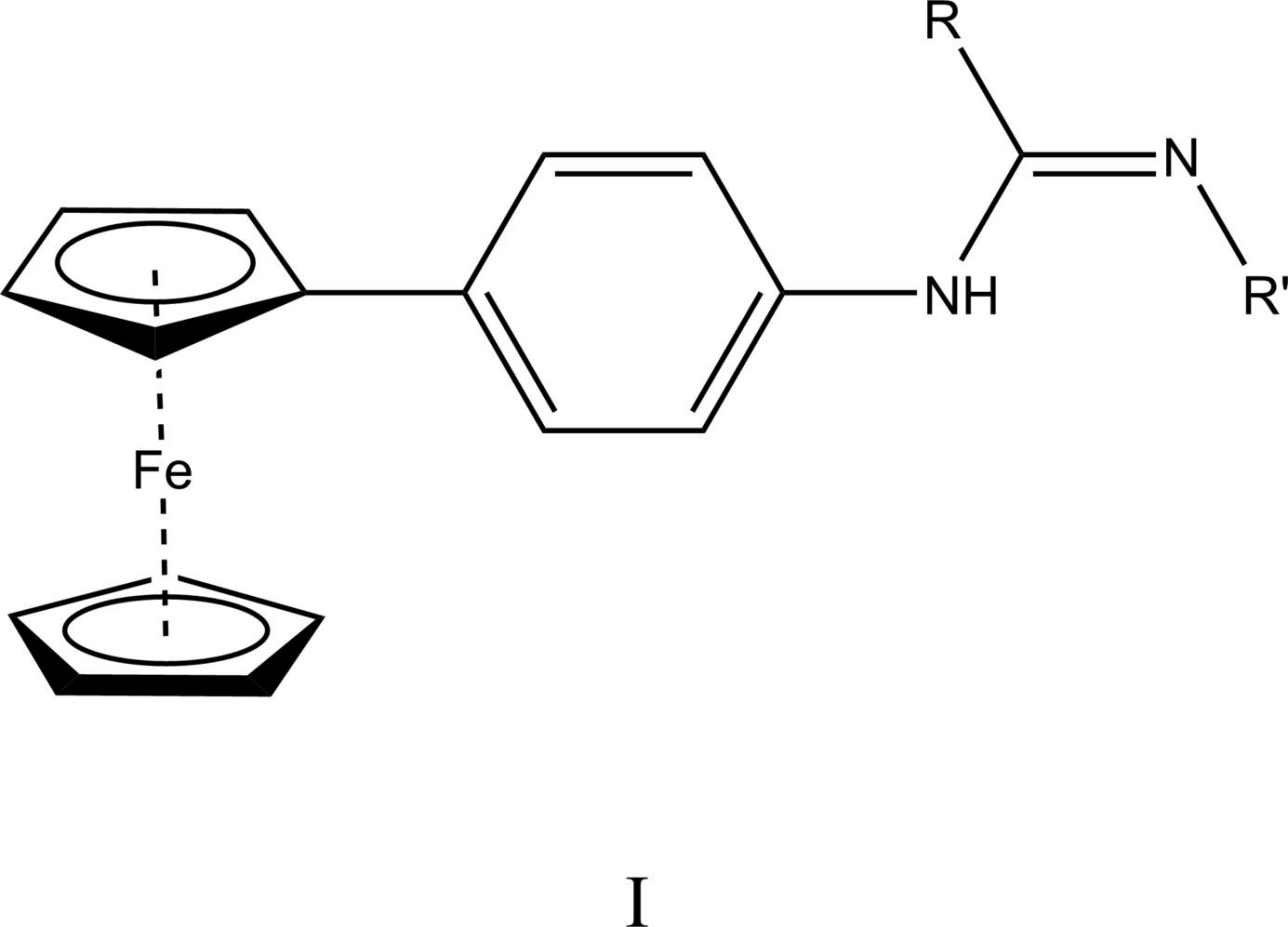


## 6. Synthesis and crystallization

4-Ferrocenyl aniline was synthesized using a previously described procedure (Adil *et al.*, 2018 ▸). In a 100 ml flask, 4-ferrocenyl aniline (1 mmol) and 4-ferrocenylphenyl carbonimidic dichloride (1 mmol) were dissolved in DMF (20 mL) to which potassium carbonate (2 mmol) and tetra-*n*-butyl ammonium bromide (0.20 mmol) were added. The reaction mixture was stirred at reflux for 12 h. The DMF was removed by rotary evaporation and distilled water was added to the residue, which was then extracted with di­chloro­methane. The organic phase was dried with Na_2_SO_4_, filtered and evaporated under reduced pressure. The residue was then purified by silica column chromatography, eluting with a mixture of hexa­ne/ethyl acetate (4/1) and the solid obtained upon evaporation of the eluant was recrystallized from ethanol (yield: 92%, m.p. 258 K). 
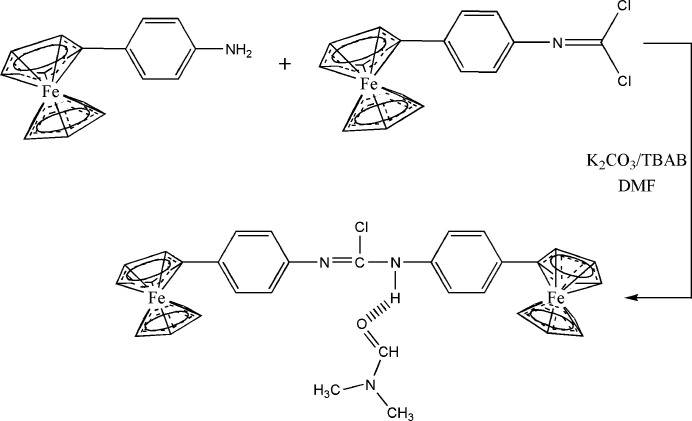


## 7. Refinement

Crystal data, data collection and structure refinement details are summarized in Table 3 ▸. Hydrogen atoms attached to carbon were placed in idealized positions with isotropic displacement parameters tied to those of the attached atoms. The two components of the disordered hydrogen attached to nitro­gen were located in a difference map and refined with a DFIX 0.91 0.01 instruction with isotropic displacement parameters 1.2 times that of the attached nitro­gen and equal occupancies.

## Supplementary Material

Crystal structure: contains datablock(s) global, I. DOI: 10.1107/S2056989024001002/ox2002sup1.cif

Structure factors: contains datablock(s) I. DOI: 10.1107/S2056989024001002/ox2002Isup2.hkl

Supporting information file. DOI: 10.1107/S2056989024001002/ox2002Isup3.cdx

CCDC reference: 2329443

Additional supporting information:  crystallographic information; 3D view; checkCIF report

## Figures and Tables

**Table 1:** Dihedral angles (°) between planes

Planes	Dihedral angle
C6–C10 *vs* C11–C16	23.37 (12)
C11–C16 *vs* N1/C17/N2/C11	45.39 (7)
N1/C17/N2/C11 *vs* C18–C23	9.09 (13)
C18–C23 *vs* C24–C28	9.08 (15)

**Table 2:** Hydrogen-bond geometry (Å, °) *Cg*2, *Cg*4, *Cg*5 and *Cg*6 are the centroids of the C6–C10, C29–C23 C11–C16 and C18–C23 rings, respectively.

*D*—H⋯*A*	*D*—H	H⋯*A*	*D*⋯*A*	*D*—H⋯*A*
N1—H1⋯O1	0.91 (1)	1.98 (2)	2.862 (3)	163 (5)
C7—H7⋯*Cg*6^i^	1.00	2.63	3.569 (3)	157
C19—H19⋯*Cg*5^ii^	0.95	2.63	3.286 (3)	126
C23—H23⋯Cl1	0.95	2.55	3.219 (2)	127
C25—H25⋯*Cg*2^ii^	1.00	2.94	3.913 (3)	163
C34—H34⋯*Cg*5^i^	0.95	2.71	3.632 (3)	164
C35—H35*C*⋯*Cg*4^iii^	0.98	2.97	3.624 (3)	125

Symmetry codes: (i) 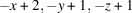 ; (ii) 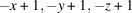 ; (iii) 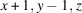 .

**Table 3:** Experimental details

Crystal data	
Chemical formula	[Fe_2_(C_5_H_5_)_2_(C_23_H_17_ClN_2_)]
*M*_r_	671.81
Crystal system, space group	Triclinic, *P* 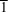
Temperature (K)	150
*a*, *b*, *c* (Å)	8.0175 (10), 11.3134 (14), 17.408 (2)
α, β, γ (°)	95.099 (2), 99.963 (2), 96.414 (2)
*V* (Å^3^)	1536.0 (3)
*Z*	2
Radiation type	Mo *K*α
μ (mm^−1^)	1.07
Crystal size (mm)	0.35 × 0.30 × 0.03

Data collection	
Diffractometer	Bruker D8 QUEST PHOTON 3 diffractometer
Absorption correction	Numerical (*SADABS*; Krause *et al.*, 2015 ▸)
*T*_min_, *T*_max_	0.71, 0.96
No. of measured, independent and observed [*I* > 2σ(*I*)] reflections	19205, 9949, 7143
*R*_int_	0.034
(sin θ/λ)_max_ (Å^−1^)	0.737

Refinement	
*R*[*F* ^2^ > 2σ(*F* ^2^)], *wR*(*F* ^2^), *S*	0.047, 0.131, 1.03
No. of reflections	9949
No. of parameters	396
No. of restraints	2
H-atom treatment	H atoms treated by a mixture of independent and constrained refinement
Δρ_max_, Δρ_min_ (e Å^−3^)	0.54, −0.85

Computer programs: *APEX4* and *SAINT* (Bruker, 2021 ▸), *SHELXT* (Sheldrick, 2015*a* ▸), *SHELXL-2019/1* (Sheldrick, 2015*b* ▸), *DIAMOND* (Brandenburg & Putz, 2012 ▸) and *SHELXTL* (Sheldrick, 2008 ▸).

## supplementary crystallographic information

### Crystal data

**Table:**

[Fe_2_(C_5_H_5_)_2_(C_23_H_17_ClN_2_)]	*Z* = 2
*M**_r_* = 671.81	*F*(000) = 696
Triclinic, *P*1	*D*_x_ = 1.453 Mg m^−^^3^
*a* = 8.0175 (10) Å	Mo *K*α radiation, λ = 0.71073 Å
*b* = 11.3134 (14) Å	Cell parameters from 9233 reflections
*c* = 17.408 (2) Å	θ = 2.3–31.6°
α = 95.099 (2)°	µ = 1.07 mm^−^^1^
β = 99.963 (2)°	*T* = 150 K
γ = 96.414 (2)°	Plate, orange
*V* = 1536.0 (3) Å^3^	0.35 × 0.30 × 0.03 mm

### Data collection

**Table:**

Bruker D8 QUEST PHOTON 3 diffractometer	9949 independent reflections
Radiation source: fine-focus sealed tube	7143 reflections with *I* > 2σ(*I*)
Graphite monochromator	*R*_int_ = 0.034
Detector resolution: 7.3910 pixels mm^-1^	θ_max_ = 31.6°, θ_min_ = 2.3°
φ and ω scans	*h* = −11→11
Absorption correction: numerical (*SADABS*; Krause *et al.*, 2015)	*k* = −16→16
*T*_min_ = 0.71, *T*_max_ = 0.96	*l* = −25→25
19205 measured reflections	

### Refinement

**Table:**

Refinement on *F*^2^	Primary atom site location: dual
Least-squares matrix: full	Secondary atom site location: difference Fourier map
*R*[*F*^2^ > 2σ(*F*^2^)] = 0.047	Hydrogen site location: mixed
*wR*(*F*^2^) = 0.131	H atoms treated by a mixture of independent and constrained refinement
*S* = 1.03	*w* = 1/[σ^2^(*F*_o_^2^) + (0.0573*P*)^2^ + 0.8875*P*] where *P* = (*F*_o_^2^ + 2*F*_c_^2^)/3
9949 reflections	(Δ/σ)_max_ = 0.001
396 parameters	Δρ_max_ = 0.54 e Å^−^^3^
2 restraints	Δρ_min_ = −0.85 e Å^−^^3^

### Special details

**Table:**

Experimental. The diffraction data were collected in three sets of 363 frames (0.5° width in ω) at φ = 0, 120 and 240°. A scan time of 20 sec/frame was used.
Geometry. All esds (except the esd in the dihedral angle between two l.s. planes) are estimated using the full covariance matrix. The cell esds are taken into account individually in the estimation of esds in distances, angles and torsion angles; correlations between esds in cell parameters are only used when they are defined by crystal symmetry. An approximate (isotropic) treatment of cell esds is used for estimating esds involving l.s. planes.
Refinement. Refinement of F^2^ against ALL reflections. The weighted R-factor wR and goodness of fit S are based on F^2^, conventional R-factors R are based on F, with F set to zero for negative F^2^. The threshold expression of F^2^ > 2sigma(F^2^) is used only for calculating R-factors(gt) etc. and is not relevant to the choice of reflections for refinement. R-factors based on F^2^ are statistically about twice as large as those based on F, and R- factors based on ALL data will be even larger. H-atoms attached to carbon were placed in calculated positions (C—H = 0.95 - 1.00 Å) and were included as riding contributions with isotropic displacement parameters 1.2 - 1.5 times those of the attached atoms. Those attached to nitrogen were placed in locations derived from a difference map and refined with a DFIX 0.91 0.01 instruction. The central {NH—C(Cl)═N} portion is disordered in essentially equal amounts leading to two locations for the hydrogen atom and equal NC distances. Two reflections affected by the beamstop were omitted from the final refinement.

### Fractional atomic coordinates and isotropic or equivalent isotropic displacement parameters (Å^2^)

**Table:**

	*x*	*y*	*z*	*U*_iso_*/*U*_eq_	Occ. (<1)
Fe1	1.37373 (4)	0.63325 (3)	0.87322 (2)	0.02325 (8)	
Fe2	0.07704 (4)	0.90503 (3)	0.14060 (2)	0.02200 (8)	
Cl1	0.81676 (8)	0.78250 (5)	0.48737 (4)	0.03526 (14)	
N1	0.7405 (2)	0.55410 (16)	0.51322 (11)	0.0235 (4)	
H1	0.695 (6)	0.4785 (19)	0.492 (3)	0.028*	0.5
N2	0.5880 (2)	0.60294 (17)	0.40311 (11)	0.0250 (4)	
H1B	0.545 (7)	0.5247 (16)	0.401 (3)	0.030*	0.5
C1	1.6276 (3)	0.6844 (3)	0.8766 (2)	0.0520 (9)	
H1A	1.719399	0.631160	0.882788	0.062*	
C2	1.5318 (4)	0.7073 (3)	0.8050 (2)	0.0458 (7)	
H2	1.543702	0.673373	0.751491	0.055*	
C3	1.4175 (3)	0.7870 (2)	0.82208 (16)	0.0360 (6)	
H3	1.333001	0.818748	0.782517	0.043*	
C4	1.4400 (3)	0.8136 (2)	0.90388 (17)	0.0360 (6)	
H4	1.375259	0.867808	0.932501	0.043*	
C5	1.5711 (4)	0.7501 (3)	0.93865 (19)	0.0453 (7)	
H5	1.616204	0.751766	0.996066	0.054*	
C6	1.1536 (3)	0.53288 (19)	0.81168 (13)	0.0218 (4)	
C7	1.2867 (3)	0.45906 (19)	0.82827 (13)	0.0233 (4)	
H7	1.334693	0.410700	0.788454	0.028*	
C8	1.3395 (3)	0.4672 (2)	0.91084 (14)	0.0286 (5)	
H8	1.431207	0.425495	0.939174	0.034*	
C9	1.2413 (3)	0.5455 (2)	0.94644 (14)	0.0290 (5)	
H9	1.251227	0.568080	1.004055	0.035*	
C10	1.1260 (3)	0.5865 (2)	0.88539 (13)	0.0249 (4)	
H10	1.041297	0.643043	0.892816	0.030*	
C11	1.0568 (3)	0.54542 (19)	0.73328 (12)	0.0213 (4)	
C12	1.0447 (3)	0.4563 (2)	0.67087 (13)	0.0233 (4)	
H12	1.105904	0.389711	0.678342	0.028*	
C13	0.9452 (3)	0.4631 (2)	0.59827 (13)	0.0241 (4)	
H13	0.939304	0.401614	0.556665	0.029*	
C14	0.8535 (3)	0.56001 (19)	0.58587 (13)	0.0223 (4)	
C15	0.8653 (3)	0.65036 (19)	0.64720 (13)	0.0243 (4)	
H15	0.804354	0.717018	0.639563	0.029*	
C16	0.9662 (3)	0.64274 (19)	0.71946 (13)	0.0238 (4)	
H16	0.974038	0.705219	0.760665	0.029*	
C17	0.7107 (3)	0.64420 (19)	0.46750 (13)	0.0224 (4)	
C18	0.5173 (3)	0.65737 (19)	0.33737 (13)	0.0215 (4)	
C19	0.3789 (3)	0.5892 (2)	0.28742 (14)	0.0260 (4)	
H19	0.339475	0.511829	0.299358	0.031*	
C20	0.2979 (3)	0.6318 (2)	0.22089 (13)	0.0255 (4)	
H20	0.203611	0.583399	0.187916	0.031*	
C21	0.3523 (3)	0.74514 (18)	0.20122 (13)	0.0209 (4)	
C22	0.4938 (3)	0.8110 (2)	0.25035 (14)	0.0267 (5)	
H22	0.535424	0.887311	0.237483	0.032*	
C23	0.5765 (3)	0.7693 (2)	0.31754 (14)	0.0272 (5)	
H23	0.672821	0.816635	0.349791	0.033*	
C24	0.2600 (3)	0.79388 (19)	0.13310 (13)	0.0216 (4)	
C25	0.0994 (3)	0.7426 (2)	0.08542 (14)	0.0255 (4)	
H25	0.033179	0.664561	0.091133	0.031*	
C26	0.0495 (3)	0.8218 (2)	0.02916 (14)	0.0277 (5)	
H26	−0.057611	0.809202	−0.011338	0.033*	
C27	0.1775 (3)	0.9230 (2)	0.04117 (14)	0.0270 (5)	
H27	0.175995	0.993803	0.010649	0.032*	
C28	0.3065 (3)	0.9062 (2)	0.10470 (13)	0.0252 (4)	
H28	0.411516	0.963704	0.126666	0.030*	
C29	−0.1556 (3)	0.9466 (2)	0.15862 (16)	0.0346 (5)	
H29	−0.265962	0.926545	0.120477	0.041*	
C30	−0.0392 (3)	1.0524 (2)	0.16516 (15)	0.0325 (5)	
H30	−0.052690	1.120135	0.132317	0.039*	
C31	0.1004 (4)	1.0450 (2)	0.22600 (15)	0.0339 (5)	
H31	0.202644	1.106623	0.243602	0.041*	
C32	0.0705 (4)	0.9344 (3)	0.25732 (15)	0.0364 (6)	
H32	0.147494	0.904772	0.300942	0.044*	
C33	−0.0878 (4)	0.8739 (3)	0.21588 (17)	0.0379 (6)	
H33	−0.142146	0.793681	0.224987	0.045*	
O1	0.5848 (2)	0.33700 (16)	0.41848 (12)	0.0377 (4)	
N3	0.6722 (3)	0.15580 (18)	0.39189 (13)	0.0327 (5)	
C34	0.6909 (3)	0.2730 (2)	0.40136 (16)	0.0350 (6)	
H34	0.797002	0.312878	0.394321	0.042*	
C35	0.8031 (3)	0.0875 (2)	0.37001 (17)	0.0354 (6)	
H35A	0.831326	0.032017	0.408975	0.053*	
H35B	0.905549	0.142311	0.367938	0.053*	
H35C	0.760721	0.042240	0.318342	0.053*	
C36	0.5091 (5)	0.0882 (3)	0.3964 (3)	0.0859 (16)	
H36A	0.528889	0.023536	0.429413	0.129*	
H36B	0.450962	0.054033	0.343533	0.129*	
H36C	0.437852	0.141506	0.419112	0.129*	

### Atomic displacement parameters (Å^2^)

**Table:**

	*U*^11^	*U*^22^	*U*^33^	*U*^12^	*U*^13^	*U*^23^
Fe1	0.01885 (15)	0.02022 (16)	0.02790 (17)	−0.00233 (11)	0.00133 (12)	−0.00034 (12)
Fe2	0.02162 (15)	0.01953 (15)	0.02435 (16)	0.00175 (11)	0.00369 (12)	0.00191 (11)
Cl1	0.0389 (3)	0.0267 (3)	0.0347 (3)	−0.0060 (2)	−0.0032 (2)	0.0059 (2)
N1	0.0256 (9)	0.0188 (8)	0.0239 (9)	0.0004 (7)	0.0005 (7)	0.0016 (7)
N2	0.0263 (9)	0.0211 (9)	0.0252 (9)	−0.0009 (7)	−0.0007 (7)	0.0049 (7)
C1	0.0189 (12)	0.0305 (14)	0.104 (3)	−0.0030 (10)	0.0082 (14)	0.0055 (16)
C2	0.0434 (16)	0.0371 (15)	0.0573 (19)	−0.0146 (12)	0.0282 (14)	−0.0042 (13)
C3	0.0358 (14)	0.0278 (12)	0.0413 (15)	−0.0080 (10)	0.0044 (11)	0.0079 (11)
C4	0.0383 (14)	0.0216 (11)	0.0458 (15)	−0.0070 (10)	0.0115 (12)	−0.0030 (10)
C5	0.0377 (15)	0.0356 (15)	0.0491 (17)	−0.0186 (12)	−0.0144 (12)	0.0045 (12)
C6	0.0190 (9)	0.0199 (9)	0.0248 (10)	−0.0025 (7)	0.0028 (8)	0.0018 (8)
C7	0.0219 (10)	0.0188 (10)	0.0266 (11)	−0.0012 (7)	0.0015 (8)	0.0004 (8)
C8	0.0279 (11)	0.0253 (11)	0.0295 (12)	−0.0006 (9)	−0.0019 (9)	0.0055 (9)
C9	0.0276 (11)	0.0316 (12)	0.0253 (11)	−0.0034 (9)	0.0033 (9)	0.0019 (9)
C10	0.0210 (10)	0.0258 (11)	0.0260 (11)	−0.0021 (8)	0.0036 (8)	0.0009 (8)
C11	0.0196 (9)	0.0210 (10)	0.0221 (10)	−0.0018 (7)	0.0031 (8)	0.0022 (8)
C12	0.0225 (10)	0.0225 (10)	0.0248 (10)	0.0034 (8)	0.0036 (8)	0.0022 (8)
C13	0.0260 (10)	0.0210 (10)	0.0234 (10)	0.0013 (8)	0.0023 (8)	−0.0010 (8)
C14	0.0219 (10)	0.0216 (10)	0.0227 (10)	−0.0009 (8)	0.0045 (8)	0.0030 (8)
C15	0.0252 (10)	0.0193 (10)	0.0282 (11)	0.0029 (8)	0.0043 (9)	0.0037 (8)
C16	0.0252 (10)	0.0181 (10)	0.0267 (11)	−0.0006 (8)	0.0044 (8)	−0.0006 (8)
C17	0.0200 (9)	0.0223 (10)	0.0244 (10)	0.0011 (8)	0.0033 (8)	0.0036 (8)
C18	0.0206 (9)	0.0207 (10)	0.0231 (10)	0.0020 (7)	0.0036 (8)	0.0029 (8)
C19	0.0237 (10)	0.0218 (10)	0.0308 (12)	−0.0042 (8)	0.0043 (9)	0.0050 (8)
C20	0.0223 (10)	0.0232 (10)	0.0278 (11)	−0.0047 (8)	0.0002 (8)	0.0036 (8)
C21	0.0195 (9)	0.0195 (9)	0.0240 (10)	0.0020 (7)	0.0050 (8)	0.0026 (8)
C22	0.0276 (11)	0.0192 (10)	0.0308 (12)	−0.0038 (8)	0.0013 (9)	0.0052 (8)
C23	0.0248 (11)	0.0229 (11)	0.0302 (12)	−0.0038 (8)	−0.0013 (9)	0.0042 (9)
C24	0.0204 (9)	0.0188 (9)	0.0261 (10)	0.0028 (7)	0.0051 (8)	0.0030 (8)
C25	0.0261 (11)	0.0196 (10)	0.0290 (11)	0.0019 (8)	0.0030 (9)	−0.0012 (8)
C26	0.0285 (11)	0.0281 (11)	0.0245 (11)	0.0048 (9)	0.0004 (9)	−0.0013 (9)
C27	0.0284 (11)	0.0282 (11)	0.0260 (11)	0.0045 (9)	0.0064 (9)	0.0075 (9)
C28	0.0224 (10)	0.0257 (11)	0.0279 (11)	0.0014 (8)	0.0056 (8)	0.0061 (9)
C29	0.0264 (12)	0.0400 (14)	0.0390 (14)	0.0102 (10)	0.0080 (10)	0.0019 (11)
C30	0.0404 (14)	0.0267 (12)	0.0328 (13)	0.0127 (10)	0.0086 (11)	0.0015 (9)
C31	0.0391 (14)	0.0301 (12)	0.0302 (12)	0.0065 (10)	0.0036 (10)	−0.0063 (10)
C32	0.0452 (15)	0.0407 (14)	0.0275 (12)	0.0175 (12)	0.0105 (11)	0.0040 (10)
C33	0.0392 (14)	0.0380 (14)	0.0431 (15)	0.0072 (11)	0.0226 (12)	0.0083 (12)
O1	0.0391 (10)	0.0233 (9)	0.0504 (11)	0.0015 (7)	0.0127 (9)	−0.0026 (8)
N3	0.0327 (11)	0.0233 (10)	0.0435 (12)	0.0002 (8)	0.0136 (9)	0.0027 (9)
C34	0.0342 (13)	0.0248 (12)	0.0447 (15)	−0.0042 (10)	0.0123 (11)	−0.0030 (10)
C35	0.0308 (13)	0.0270 (12)	0.0465 (15)	0.0022 (10)	0.0055 (11)	−0.0014 (11)
C36	0.061 (2)	0.0351 (18)	0.173 (5)	−0.0037 (16)	0.064 (3)	0.000 (2)

### Geometric parameters (Å, º)

**Table:**

Fe1—C1	2.042 (3)	C12—C13	1.386 (3)
Fe1—C2	2.044 (3)	C12—H12	0.9500
Fe1—C3	2.046 (2)	C13—C14	1.398 (3)
Fe1—C5	2.047 (3)	C13—H13	0.9500
Fe1—C4	2.048 (2)	C14—C15	1.394 (3)
Fe1—C9	2.048 (2)	C15—C16	1.388 (3)
Fe1—C8	2.049 (2)	C15—H15	0.9500
Fe1—C10	2.049 (2)	C16—H16	0.9500
Fe1—C7	2.053 (2)	C18—C19	1.392 (3)
Fe1—C6	2.058 (2)	C18—C23	1.396 (3)
Fe2—C25	2.038 (2)	C19—C20	1.380 (3)
Fe2—C32	2.040 (3)	C19—H19	0.9500
Fe2—C33	2.041 (3)	C20—C21	1.399 (3)
Fe2—C28	2.042 (2)	C20—H20	0.9500
Fe2—C31	2.042 (2)	C21—C22	1.391 (3)
Fe2—C29	2.043 (2)	C21—C24	1.470 (3)
Fe2—C26	2.044 (2)	C22—C23	1.389 (3)
Fe2—C30	2.047 (2)	C22—H22	0.9500
Fe2—C27	2.048 (2)	C23—H23	0.9500
Fe2—C24	2.050 (2)	C24—C25	1.436 (3)
Cl1—C17	1.672 (2)	C24—C28	1.438 (3)
N1—C17	1.365 (3)	C25—C26	1.423 (3)
N1—C14	1.414 (3)	C25—H25	1.0000
N1—H1	0.908 (10)	C26—C27	1.424 (3)
N2—C17	1.366 (3)	C26—H26	1.0000
N2—C18	1.410 (3)	C27—C28	1.419 (3)
N2—H1B	0.909 (10)	C27—H27	1.0000
C1—C2	1.407 (5)	C28—H28	1.0000
C1—C5	1.423 (5)	C29—C30	1.417 (4)
C1—H1A	1.0000	C29—C33	1.423 (4)
C2—C3	1.404 (4)	C29—H29	1.0000
C2—H2	1.0000	C30—C31	1.417 (4)
C3—C4	1.405 (4)	C30—H30	1.0000
C3—H3	1.0000	C31—C32	1.420 (4)
C4—C5	1.419 (4)	C31—H31	1.0000
C4—H4	1.0000	C32—C33	1.414 (4)
C5—H5	1.0000	C32—H32	1.0000
C6—C10	1.433 (3)	C33—H33	1.0000
C6—C7	1.434 (3)	O1—C34	1.234 (3)
C6—C11	1.475 (3)	N3—C34	1.310 (3)
C7—C8	1.418 (3)	N3—C35	1.452 (3)
C7—H7	1.0000	N3—C36	1.457 (4)
C8—C9	1.420 (4)	C34—H34	0.9500
C8—H8	1.0000	C35—H35A	0.9800
C9—C10	1.429 (3)	C35—H35B	0.9800
C9—H9	1.0000	C35—H35C	0.9800
C10—H10	1.0000	C36—H36A	0.9800
C11—C12	1.398 (3)	C36—H36B	0.9800
C11—C16	1.401 (3)	C36—H36C	0.9800
			
C1—Fe1—C2	40.28 (14)	Fe1—C7—H7	125.9
C1—Fe1—C3	67.51 (12)	C7—C8—C9	108.5 (2)
C2—Fe1—C3	40.15 (12)	C7—C8—Fe1	69.91 (13)
C1—Fe1—C5	40.74 (14)	C9—C8—Fe1	69.71 (14)
C2—Fe1—C5	68.17 (13)	C7—C8—H8	125.8
C3—Fe1—C5	67.77 (11)	C9—C8—H8	125.8
C1—Fe1—C4	68.04 (12)	Fe1—C8—H8	125.8
C2—Fe1—C4	67.86 (11)	C8—C9—C10	107.9 (2)
C3—Fe1—C4	40.13 (11)	C8—C9—Fe1	69.75 (14)
C5—Fe1—C4	40.54 (12)	C10—C9—Fe1	69.61 (13)
C1—Fe1—C9	133.24 (13)	C8—C9—H9	126.0
C2—Fe1—C9	172.21 (12)	C10—C9—H9	126.0
C3—Fe1—C9	146.77 (11)	Fe1—C9—H9	126.0
C5—Fe1—C9	109.48 (11)	C9—C10—C6	108.1 (2)
C4—Fe1—C9	115.59 (11)	C9—C10—Fe1	69.56 (13)
C1—Fe1—C8	109.76 (11)	C6—C10—Fe1	69.91 (12)
C2—Fe1—C8	132.96 (12)	C9—C10—H10	126.0
C3—Fe1—C8	171.90 (11)	C6—C10—H10	126.0
C5—Fe1—C8	115.58 (11)	Fe1—C10—H10	126.0
C4—Fe1—C8	146.98 (11)	C12—C11—C16	117.50 (19)
C9—Fe1—C8	40.54 (10)	C12—C11—C6	120.7 (2)
C1—Fe1—C10	172.56 (13)	C16—C11—C6	121.8 (2)
C2—Fe1—C10	146.10 (12)	C13—C12—C11	121.3 (2)
C3—Fe1—C10	115.30 (10)	C13—C12—H12	119.4
C5—Fe1—C10	132.83 (12)	C11—C12—H12	119.4
C4—Fe1—C10	109.28 (10)	C12—C13—C14	120.5 (2)
C9—Fe1—C10	40.83 (9)	C12—C13—H13	119.8
C8—Fe1—C10	68.41 (10)	C14—C13—H13	119.8
C1—Fe1—C7	115.15 (11)	C15—C14—C13	119.1 (2)
C2—Fe1—C7	109.26 (11)	C15—C14—N1	123.6 (2)
C3—Fe1—C7	132.87 (10)	C13—C14—N1	117.03 (19)
C5—Fe1—C7	146.57 (11)	C16—C15—C14	119.9 (2)
C4—Fe1—C7	171.80 (10)	C16—C15—H15	120.1
C9—Fe1—C7	68.31 (10)	C14—C15—H15	120.1
C8—Fe1—C7	40.45 (9)	C15—C16—C11	121.8 (2)
C10—Fe1—C7	68.51 (9)	C15—C16—H16	119.1
C1—Fe1—C6	146.01 (12)	C11—C16—H16	119.1
C2—Fe1—C6	114.71 (11)	N1—C17—N2	109.99 (19)
C3—Fe1—C6	109.19 (10)	N1—C17—Cl1	123.90 (17)
C5—Fe1—C6	171.99 (12)	N2—C17—Cl1	126.07 (17)
C4—Fe1—C6	132.48 (10)	C19—C18—C23	118.5 (2)
C9—Fe1—C6	68.68 (9)	C19—C18—N2	115.36 (19)
C8—Fe1—C6	68.48 (9)	C23—C18—N2	126.1 (2)
C10—Fe1—C6	40.84 (9)	C20—C19—C18	121.3 (2)
C7—Fe1—C6	40.83 (9)	C20—C19—H19	119.4
C25—Fe2—C32	123.61 (10)	C18—C19—H19	119.4
C25—Fe2—C33	106.62 (11)	C19—C20—C21	121.1 (2)
C32—Fe2—C33	40.56 (12)	C19—C20—H20	119.5
C25—Fe2—C28	68.76 (9)	C21—C20—H20	119.5
C32—Fe2—C28	119.74 (11)	C22—C21—C20	117.1 (2)
C33—Fe2—C28	154.51 (11)	C22—C21—C24	121.61 (19)
C25—Fe2—C31	160.99 (10)	C20—C21—C24	121.25 (19)
C32—Fe2—C31	40.72 (11)	C23—C22—C21	122.4 (2)
C33—Fe2—C31	68.25 (11)	C23—C22—H22	118.8
C28—Fe2—C31	107.53 (10)	C21—C22—H22	118.8
C25—Fe2—C29	120.65 (10)	C22—C23—C18	119.6 (2)
C32—Fe2—C29	68.48 (11)	C22—C23—H23	120.2
C33—Fe2—C29	40.78 (11)	C18—C23—H23	120.2
C28—Fe2—C29	163.14 (10)	C25—C24—C28	106.56 (19)
C31—Fe2—C29	68.25 (11)	C25—C24—C21	126.56 (19)
C25—Fe2—C26	40.80 (9)	C28—C24—C21	126.74 (19)
C32—Fe2—C26	161.42 (11)	C25—C24—Fe2	68.99 (12)
C33—Fe2—C26	125.31 (11)	C28—C24—Fe2	69.14 (12)
C28—Fe2—C26	68.49 (9)	C21—C24—Fe2	123.44 (15)
C31—Fe2—C26	156.89 (10)	C26—C25—C24	108.57 (19)
C29—Fe2—C26	108.59 (10)	C26—C25—Fe2	69.84 (13)
C25—Fe2—C30	156.43 (10)	C24—C25—Fe2	69.87 (12)
C32—Fe2—C30	68.44 (10)	C26—C25—H25	125.7
C33—Fe2—C30	68.33 (11)	C24—C25—H25	125.7
C28—Fe2—C30	125.81 (10)	Fe2—C25—H25	125.7
C31—Fe2—C30	40.55 (10)	C25—C26—C27	108.1 (2)
C29—Fe2—C30	40.53 (11)	C25—C26—Fe2	69.37 (13)
C26—Fe2—C30	122.09 (10)	C27—C26—Fe2	69.78 (13)
C25—Fe2—C27	68.68 (10)	C25—C26—H26	125.9
C32—Fe2—C27	155.59 (11)	C27—C26—H26	125.9
C33—Fe2—C27	163.08 (11)	Fe2—C26—H26	125.9
C28—Fe2—C27	40.62 (9)	C28—C27—C26	107.9 (2)
C31—Fe2—C27	121.45 (11)	C28—C27—Fe2	69.47 (13)
C29—Fe2—C27	126.45 (10)	C26—C27—Fe2	69.50 (13)
C26—Fe2—C27	40.72 (9)	C28—C27—H27	126.0
C30—Fe2—C27	109.09 (10)	C26—C27—H27	126.0
C25—Fe2—C24	41.13 (9)	Fe2—C27—H27	126.0
C32—Fe2—C24	105.37 (10)	C27—C28—C24	108.8 (2)
C33—Fe2—C24	118.80 (10)	C27—C28—Fe2	69.91 (13)
C28—Fe2—C24	41.16 (8)	C24—C28—Fe2	69.71 (12)
C31—Fe2—C24	123.86 (10)	C27—C28—H28	125.6
C29—Fe2—C24	154.87 (10)	C24—C28—H28	125.6
C26—Fe2—C24	69.09 (9)	Fe2—C28—H28	125.6
C30—Fe2—C24	161.86 (10)	C30—C29—C33	107.9 (2)
C27—Fe2—C24	69.09 (9)	C30—C29—Fe2	69.92 (14)
C17—N1—C14	128.02 (18)	C33—C29—Fe2	69.55 (14)
C17—N1—H1	117 (3)	C30—C29—H29	126.0
C14—N1—H1	114 (3)	C33—C29—H29	126.0
C17—N2—C18	132.46 (19)	Fe2—C29—H29	126.0
C17—N2—H1B	114 (3)	C29—C30—C31	107.9 (2)
C18—N2—H1B	113 (3)	C29—C30—Fe2	69.55 (14)
C2—C1—C5	108.2 (3)	C31—C30—Fe2	69.53 (14)
C2—C1—Fe1	69.94 (15)	C29—C30—H30	126.0
C5—C1—Fe1	69.83 (16)	C31—C30—H30	126.0
C2—C1—H1A	125.9	Fe2—C30—H30	126.0
C5—C1—H1A	125.9	C30—C31—C32	108.2 (2)
Fe1—C1—H1A	125.9	C30—C31—Fe2	69.92 (14)
C3—C2—C1	107.8 (3)	C32—C31—Fe2	69.56 (14)
C3—C2—Fe1	70.00 (15)	C30—C31—H31	125.9
C1—C2—Fe1	69.79 (17)	C32—C31—H31	125.9
C3—C2—H2	126.1	Fe2—C31—H31	125.9
C1—C2—H2	126.1	C33—C32—C31	107.8 (2)
Fe1—C2—H2	126.1	C33—C32—Fe2	69.75 (15)
C2—C3—C4	108.8 (3)	C31—C32—Fe2	69.72 (14)
C2—C3—Fe1	69.85 (16)	C33—C32—H32	126.1
C4—C3—Fe1	70.02 (15)	C31—C32—H32	126.1
C2—C3—H3	125.6	Fe2—C32—H32	126.1
C4—C3—H3	125.6	C32—C33—C29	108.1 (2)
Fe1—C3—H3	125.6	C32—C33—Fe2	69.70 (15)
C3—C4—C5	107.8 (3)	C29—C33—Fe2	69.67 (14)
C3—C4—Fe1	69.85 (14)	C32—C33—H33	125.9
C5—C4—Fe1	69.69 (15)	C29—C33—H33	125.9
C3—C4—H4	126.1	Fe2—C33—H33	125.9
C5—C4—H4	126.1	C34—N3—C35	123.0 (2)
Fe1—C4—H4	126.1	C34—N3—C36	120.0 (2)
C4—C5—C1	107.3 (3)	C35—N3—C36	116.8 (2)
C4—C5—Fe1	69.77 (14)	O1—C34—N3	126.7 (2)
C1—C5—Fe1	69.44 (15)	O1—C34—H34	116.6
C4—C5—H5	126.4	N3—C34—H34	116.6
C1—C5—H5	126.4	N3—C35—H35A	109.5
Fe1—C5—H5	126.4	N3—C35—H35B	109.5
C10—C6—C7	107.30 (19)	H35A—C35—H35B	109.5
C10—C6—C11	126.6 (2)	N3—C35—H35C	109.5
C7—C6—C11	126.0 (2)	H35A—C35—H35C	109.5
C10—C6—Fe1	69.26 (12)	H35B—C35—H35C	109.5
C7—C6—Fe1	69.39 (12)	N3—C36—H36A	109.5
C11—C6—Fe1	129.10 (15)	N3—C36—H36B	109.5
C8—C7—C6	108.2 (2)	H36A—C36—H36B	109.5
C8—C7—Fe1	69.64 (13)	N3—C36—H36C	109.5
C6—C7—Fe1	69.79 (12)	H36A—C36—H36C	109.5
C8—C7—H7	125.9	H36B—C36—H36C	109.5
C6—C7—H7	125.9		
			
C5—C1—C2—C3	0.4 (3)	C18—N2—C17—Cl1	0.8 (4)
Fe1—C1—C2—C3	59.91 (18)	C17—N2—C18—C19	−173.0 (2)
C5—C1—C2—Fe1	−59.55 (19)	C17—N2—C18—C23	9.1 (4)
C1—C2—C3—C4	−0.4 (3)	C23—C18—C19—C20	−1.9 (3)
Fe1—C2—C3—C4	59.35 (18)	N2—C18—C19—C20	−179.9 (2)
C1—C2—C3—Fe1	−59.77 (18)	C18—C19—C20—C21	0.1 (4)
C2—C3—C4—C5	0.3 (3)	C19—C20—C21—C22	1.7 (3)
Fe1—C3—C4—C5	59.57 (17)	C19—C20—C21—C24	−176.2 (2)
C2—C3—C4—Fe1	−59.25 (18)	C20—C21—C22—C23	−1.8 (3)
C3—C4—C5—C1	−0.1 (3)	C24—C21—C22—C23	176.1 (2)
Fe1—C4—C5—C1	59.57 (18)	C21—C22—C23—C18	0.0 (4)
C3—C4—C5—Fe1	−59.67 (17)	C19—C18—C23—C22	1.8 (3)
C2—C1—C5—C4	−0.2 (3)	N2—C18—C23—C22	179.6 (2)
Fe1—C1—C5—C4	−59.78 (18)	C22—C21—C24—C25	−170.1 (2)
C2—C1—C5—Fe1	59.62 (18)	C20—C21—C24—C25	7.7 (3)
C10—C6—C7—C8	0.1 (2)	C22—C21—C24—C28	5.0 (3)
C11—C6—C7—C8	−176.8 (2)	C20—C21—C24—C28	−177.1 (2)
Fe1—C6—C7—C8	59.21 (15)	C22—C21—C24—Fe2	−82.7 (2)
C10—C6—C7—Fe1	−59.10 (14)	C20—C21—C24—Fe2	95.1 (2)
C11—C6—C7—Fe1	124.0 (2)	C28—C24—C25—C26	0.1 (3)
C6—C7—C8—C9	−0.1 (3)	C21—C24—C25—C26	176.1 (2)
Fe1—C7—C8—C9	59.25 (16)	Fe2—C24—C25—C26	59.31 (16)
C6—C7—C8—Fe1	−59.31 (15)	C28—C24—C25—Fe2	−59.19 (15)
C7—C8—C9—C10	0.0 (3)	C21—C24—C25—Fe2	116.8 (2)
Fe1—C8—C9—C10	59.35 (16)	C24—C25—C26—C27	−0.1 (3)
C7—C8—C9—Fe1	−59.37 (16)	Fe2—C25—C26—C27	59.21 (17)
C8—C9—C10—C6	0.1 (3)	C24—C25—C26—Fe2	−59.33 (16)
Fe1—C9—C10—C6	59.53 (15)	C25—C26—C27—C28	0.1 (3)
C8—C9—C10—Fe1	−59.44 (16)	Fe2—C26—C27—C28	59.03 (16)
C7—C6—C10—C9	−0.1 (2)	C25—C26—C27—Fe2	−58.95 (16)
C11—C6—C10—C9	176.7 (2)	C26—C27—C28—C24	0.0 (3)
Fe1—C6—C10—C9	−59.31 (15)	Fe2—C27—C28—C24	59.04 (16)
C7—C6—C10—Fe1	59.19 (14)	C26—C27—C28—Fe2	−59.04 (17)
C11—C6—C10—Fe1	−124.0 (2)	C25—C24—C28—C27	−0.1 (3)
C10—C6—C11—C12	−153.8 (2)	C21—C24—C28—C27	−176.0 (2)
C7—C6—C11—C12	22.5 (3)	Fe2—C24—C28—C27	−59.17 (16)
Fe1—C6—C11—C12	114.2 (2)	C25—C24—C28—Fe2	59.10 (15)
C10—C6—C11—C16	22.6 (3)	C21—C24—C28—Fe2	−116.8 (2)
C7—C6—C11—C16	−161.1 (2)	C33—C29—C30—C31	0.2 (3)
Fe1—C6—C11—C16	−69.4 (3)	Fe2—C29—C30—C31	−59.14 (18)
C16—C11—C12—C13	−0.8 (3)	C33—C29—C30—Fe2	59.39 (17)
C6—C11—C12—C13	175.8 (2)	C29—C30—C31—C32	−0.1 (3)
C11—C12—C13—C14	−0.2 (3)	Fe2—C30—C31—C32	−59.22 (17)
C12—C13—C14—C15	0.8 (3)	C29—C30—C31—Fe2	59.16 (17)
C12—C13—C14—N1	−173.2 (2)	C30—C31—C32—C33	−0.1 (3)
C17—N1—C14—C15	46.0 (3)	Fe2—C31—C32—C33	−59.58 (18)
C17—N1—C14—C13	−140.3 (2)	C30—C31—C32—Fe2	59.45 (17)
C13—C14—C15—C16	−0.4 (3)	C31—C32—C33—C29	0.3 (3)
N1—C14—C15—C16	173.2 (2)	Fe2—C32—C33—C29	−59.28 (18)
C14—C15—C16—C11	−0.6 (3)	C31—C32—C33—Fe2	59.56 (18)
C12—C11—C16—C15	1.2 (3)	C30—C29—C33—C32	−0.3 (3)
C6—C11—C16—C15	−175.3 (2)	Fe2—C29—C33—C32	59.29 (18)
C14—N1—C17—N2	−177.9 (2)	C30—C29—C33—Fe2	−59.62 (18)
C14—N1—C17—Cl1	4.2 (3)	C35—N3—C34—O1	178.6 (3)
C18—N2—C17—N1	−177.0 (2)	C36—N3—C34—O1	4.2 (5)

### Hydrogen-bond geometry (Å, º)

*Cg*2, *Cg*4, *Cg*5 and *Cg*6 are the centroids of the C6–C10, C29–C23 C11–C16 and C18–C23 rings, respectively.

**Table:**

*D*—H···*A*	*D*—H	H···*A*	*D*···*A*	*D*—H···*A*
N1—H1···O1	0.91 (1)	1.98 (2)	2.862 (3)	163 (5)
C7—H7···*Cg*6^i^	1.00	2.63	3.569 (3)	157
C19—H19···*Cg*5^ii^	0.95	2.63	3.286 (3)	126
C23—H23···Cl1	0.95	2.55	3.219 (2)	127
C25—H25···*Cg*2^ii^	1.00	2.94	3.913 (3)	163
C34—H34···*Cg*5^i^	0.95	2.71	3.632 (3)	164
C35—H35*C*···*Cg*4^iii^	0.98	2.97	3.624 (3)	125

Symmetry codes: (i) −*x*+2, −*y*+1, −*z*+1; (ii) −*x*+1, −*y*+1, −*z*+1; (iii) *x*+1, *y*−1, *z*.
